# Rheological Behavior of SiO_2_ Ceramic Slurry in Stereolithography and Its Prediction Model Based on POA-DELM

**DOI:** 10.3390/ma17174270

**Published:** 2024-08-29

**Authors:** Jie Zhang, Byung-Won Min, Hai Gu, Guoqing Wu, Weiwei Wu

**Affiliations:** 1 School of Mechanical Engineering, Nantong Institute of Technology, Nantong 226002, China; 2 Jiangsu Key Laboratory of 3D Printing Equipment and Application Technology, Nantong Institute of Technology, Nantong 226002, China; 3 Department of IT Engineering, Mokwon University, Daejeon 35349, Republic of Korea; 4 School of Mechanical Engineering, Yangzhou University, Yangzhou 225127, China

**Keywords:** viscosity, SiO_2_, stereolithography, POA-DELM modeling

## Abstract

Ceramic slurry is the raw material used in stereolithography, and its performance determines the printing quality. Rheological behavior, one of the most important physical factors in stereolithography, is critical in ceramic printing, significantly affecting the flow, spreading, and printing processes. The rheological behavior of SiO_2_ slurry used in stereolithography technology is investigated in the current research using different powder diameters and temperatures. The results present the apparent non-Newtonian behavior. The yielding characteristics occur in all cases. For single-powder cases, the viscosity decreases when the powder diameter is increased. When the nano-sized and micro-sized powders are mixed in different proportions, a more significant proportion of micron-sized powders will decrease the viscosity. With an increase in the nano-sized powders, the slurry exhibits the shear thinning behavior; otherwise, the shear thickening behavior is observed. Thus, the prediction model is built based on the use of the pelican optimization algorithm-deep extreme learning machine (POA-DELM), and the model in then compared with the fitted and traditional models to validate the effectiveness of the method. A more accurate viscosity prediction model will contribute to better fluid dynamic simulation in future work.

## 1. Introduction

Stereolithography is a common additive manufacturing technology, in which the photosensitive resin is selectively solidified by ultraviolet light [1,2,3]. The resulting manufactured part exhibits good accuracy and mechanical performance. In recent years, it has been considered a new 3D printing technology for use in molding hard-to-machine ceramic materials, also solving the difficulty of processing parts with complicated structures [4,5,6].

Material preparation is a crucial area of technology, and it determines the success of printing. The resin and ceramic powders are mixed in a certain proportion in ceramic stereolithography technology. A high solid content and low viscosity are expected for the mixed slurry; however, the high solid content tends to increase the viscosity. Many researchers have explored the slurry preparation process to determine the potential theory behind this mixture. Ding, G. J. et al. investigated the slurry and stability of SiC ceramic slurry, and it was found that the resin monomers (HDDA, TMPTA), dispersants, particle size, solid loading, and ball milling time are the essential impacting factors [7]. Wu, X. Q. et al. pointed out that developing low-cost Si_3_N_4_ materials based on the ACMO and HDDA resin monomers can contribute to the spread of the technology [8]. Tang, J. et al. proposed a novel strategy to prepare a SiO_2_/SiC slurry with high solid content using HDDA, TMPTA, and urethane acrylic resins [9]. Zhang, K. Q. et al. found that solid loading affects the properties of zirconia ceramic slurry with HDDA and TMPTA [10]. Li, K. H. and Zhao, Z. experimentally found that the different formulations would lead to various alumina HDDA-based suspension rheology behaviors [11]. Xing, H. Y. et al. investigated the effects of different kinds of premixed resins (ACMO, HDDA, and NPG2PODA) on the rheological behavior of an Al_2_O_3_-based suspension [12]. Li, X. B. et al. studied the effects of monomer type, coarse silicon powder, solid loading, and ambient temperature on rheological behavior. They found that the mentioned factors affect the rheological behavior of the Si_3_N_4_ ceramics [13]. The above research indicates that the formulation of the materials determines the rheological behavior of the ceramic suspension, and the rheological behavior will significantly affect the printing performance. The rheological behavior of SiO_2_ ceramic slurry is investigated, and the powder diameter and temperature are the main factors considered.

The research on rheological behavior aims to investigate the relationship between it and printing performance. However, when the mechanics problems involved in the flow and spreading processes must be analyzed, the rheological model should be obtained from the rheological behavior analysis. Wu, W. W. et al. found that the SiC paste used in direct ink writing technology is a Carreau fluid [14,15]. They also determined that the alumina slurry for stereolithography exhibits the characteristics of Sisko fluid [16]. Zhang, K. X. et al. prepared the alumina paste and found that it displays shear thinning behavior and is a power-law fluid [17]. The alumina slurry prepared by He, L. and Song, X. exhibited the apparent yielding behavior, in accordance with the Bingham or Herschel–Bulkley models [18].

In traditional research, the common technique is to fit the rheological model with the familiar models, based on the viscosity data. However, it is found that the fitted model does coincide well with the actual data in certain shear rate ranges. It is essential to develop a more accurate method to decrease the error and improve the accuracy of the fluid dynamic analysis. In recent years, artificial intelligence algorithms have become popular in many fields for achieving predictions, and these are used here to complete accurate viscosity predictions for the subsequent fluid flow analysis.

To fully understand the rheological behavior of SiO_2_ ceramic slurry, the rheological measurements are conducted using different powder sizes and temperatures, revealing the rheological characteristics. Then, an intelligent algorithm, POA-DELM, is introduced to build accurate prediction models for the viscosities; this algorithm can prevent the need for complicated rheological experiments in the future and provide viscosity–shear rate models for the fluid dynamic analysis.

This paper is organized as follows. In Section 2, the method of preparing the SiO_2_ ceramic slurry using different powder diameters is presented. The rheological experiment is also described. In Section 3, the preliminary rheological behavior is analyzed. In Section 4, the POA-DELM is proposed to predict the viscosity, and the comparisons with the actual data, the fitted model, and the traditional model are performed. Section 5 and Section 6 comprise the discussion and the conclusion.

## 2. Preparation and Rheological Experiment

### 2.1. Materials

Four kinds of amorphous silica powders, with different particle sizes, are selected as solid-phase materials, among which the nano-sized silica powder is Aerosil OX50, produced by Degussa AG Company in Germany, with a particle size ranging from 40 to 80 nm. The sub-micron silica powder is produced by Rongbai New Material Company in China, with an average particle size of 0.30 μm. The micron silica powder is produced by Douwar Company in China, with an average particle size of 3 μm. The nano- and micron-sized solid-phase materials are mixed using the two corresponding materials, in proportion. Polyethylene glycol diacrylate (PEG200DA) and hydroxyethyl acrylate (2-HEA) are selected as two monomers, among which PEG200DA is a bifunctional monomer, and the latter is a mono-acrylate. These two monomers represent similar refractive indexes to those of amorphous silica, which could reduce the van der Waals force during mixing. At the same time, these two monomers are Newtonian fluids, with a viscosity value ranging from 0.01 to 0.025 Pa·s. Adding bifunctional monomers to mono-acrylate could balance the viscosity of the slurry, endowing the final formed part with better mechanical properties. The experiments finally determined the volume percentages of the two monomers as 7% PEG200DA and 93% 2-HEA (the mixed monomer is called PEG200DA/2-HEA, as listed below). Ammonium polyacrylate (produced by Kaiwei Chemical Company, Tianjin, China) is used as the dispersant.

### 2.2. Rheological Experiment

Firstly, the slurry is prepared by slowly adding silica powder with different particle sizes into the PEG200DA/2-HEA monomer to form the mixture. After the addition, 5 wt% ammonium polyacrylate is added to the above mixture, and ball milling is conducted, with a rotating speed of 800 rpm for 2 h using a QM-3SP2 device (Nanjing University Instrument Plant, Nanjing, China). Finally, the ultrasonic method is used to remove any potential bubbles. The addition ratio of silica powder is 45% [8,9,10,11]; i.e., the volume fraction of solid phase content is 45%. Based on different particle sizes, the following six cases are investigated: nano-sized (case 1), sub-micron-sized (case 2), micron-sized (case 3), 40% nano-sized and 5% micron-sized (case 4), 25% nano-sized and 20% micron-sized (case 5), and 30% nano-sized and 15% micron-sized (case 6). After it is thoroughly mixed, the viscosity of the mixed slurry is measured using a rotary viscometer (Rheolab MC1) to determine its rheological properties. In the experiment, to understand the effect of experimental temperature on the rheological properties, the experimental temperature range selected is from 20 to 45 °C, the shear rate range is from 20 to 290 s^−1^, and 220 data groups are recorded for each case.

## 3. Rheological Results

**Figure 1:**
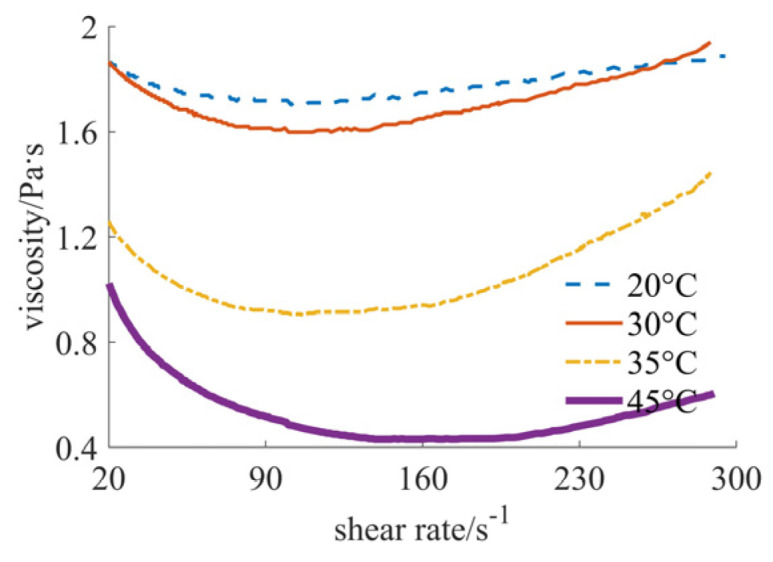
Viscosity curves of nano case.

In Figure 1, the variation in viscosity value with shear rate at different temperatures is shown, using nano-silica powder as the solid phase material. The viscosity value decreases with the increase in temperature. When the temperature is within 20–45 °C, the viscosity value ranges from 2 to 0.4 Pa·s. When the temperature is 20 °C, the slurry presents the characteristics of Newtonian fluid, or a slightly shear thinning fluid. With the increase in temperature, the slurry first reflects the characteristics of shear thinning. When the shear rate is higher than 140 s^−1^, the slurry gradually reflects the characteristics of shear thickening, which may be caused by the stronger bond between the powder particles and the resin monomers when the mixing becomes more vigorous. The higher the temperature, the more obvious the characteristics of shear thinning or shear thickening. The main reason for this result may be that polymerization starts at higher temperatures.

**Figure 2:**
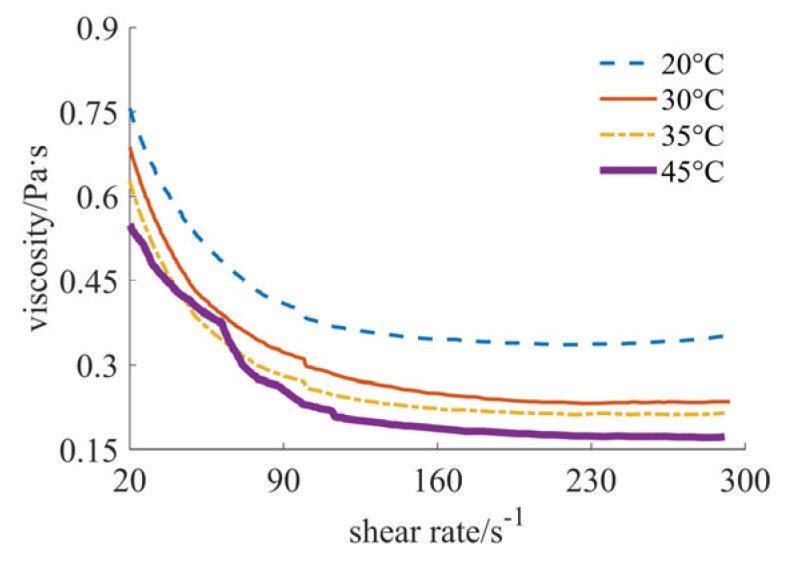
Viscosity curves of sub-micron case.

In Figure 2, the variation in viscosity value with the shear rate at different temperatures is shown, with sub-micron silicon dioxide powder as the solid phase material. As can be seen from Figure 2, the fluid at this time reflects the characteristics of shear thinning at a lower shear rate. At four different temperatures, the viscosity value of the fluid decreases with the increase in shear rate, and the viscosity value decreases with the increase in temperature. When the shear rate gradually increases to 170 s^−1^, the viscosity value curve is relatively flat, and the fluid will progressively display the characteristics of Newtonian fluid. The size range of the viscosity value is 0.14–0.8 Pa·s. At the same shear rate, the lower the temperature, the larger the viscosity value.

**Figure 3:**
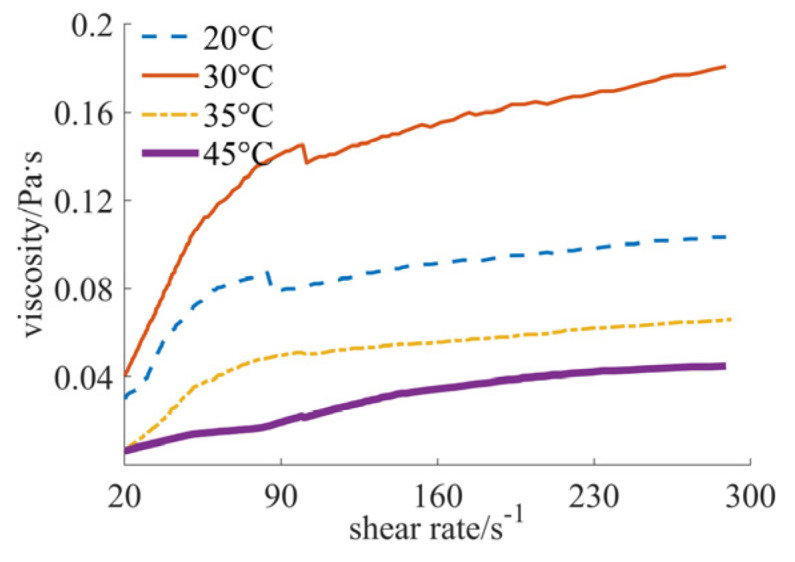
Viscosity curves of micron case.

In Figure 3, the variation curve of the viscosity value with the shear rate at different temperatures is shown, employing micron-sized silicon dioxide powder as the solid phase material. As can be seen from Figure 3, the fluid at this time obviously reflects the characteristics of shear thickening. At four different temperatures, the viscosity value of the fluid increases with the increase in shear rate, and the higher the temperature, the faster the viscosity value increases. The size range of the viscosity value is 0.005–0.2 Pa·s. At the same shear rate, the higher the temperature, the smaller the viscosity value. In addition, it can be seen in Figure 3 that the sudden change occurs at temperatures of 20 °C and 30 °C. The possible reason is that the synergism of the certain shear rate and the viscosity causes the phase transition, affecting the inner structure of the slurry [19,20].

**Figure 4:**
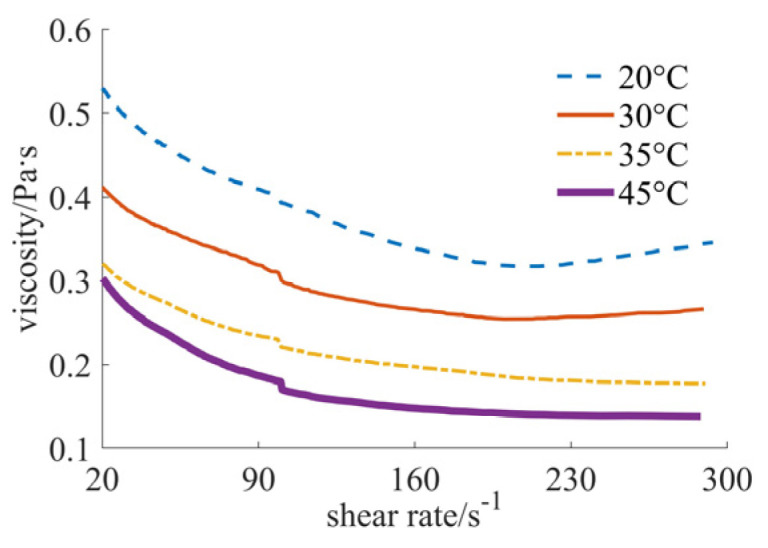
Viscosity curves of 40% nano and 5% micron case.

**Figure 5:**
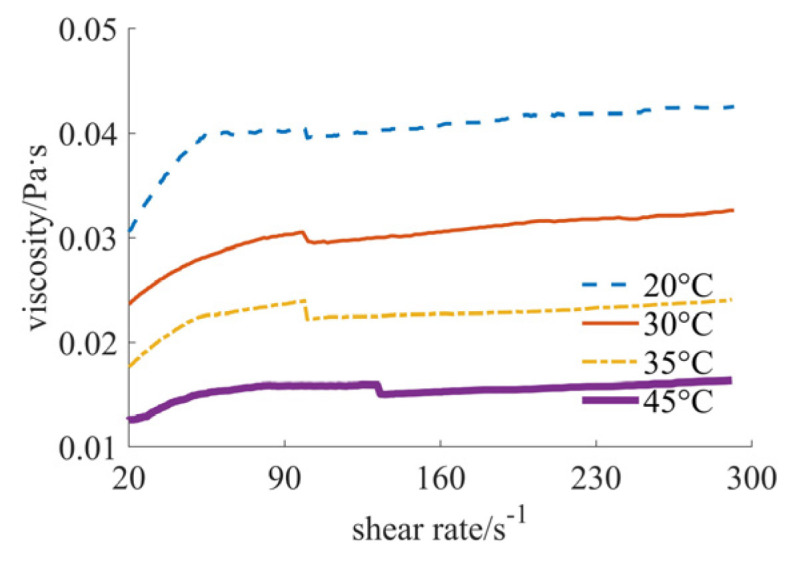
Viscosity curves of 30% nano and 15% micron case.

**Figure 6:**
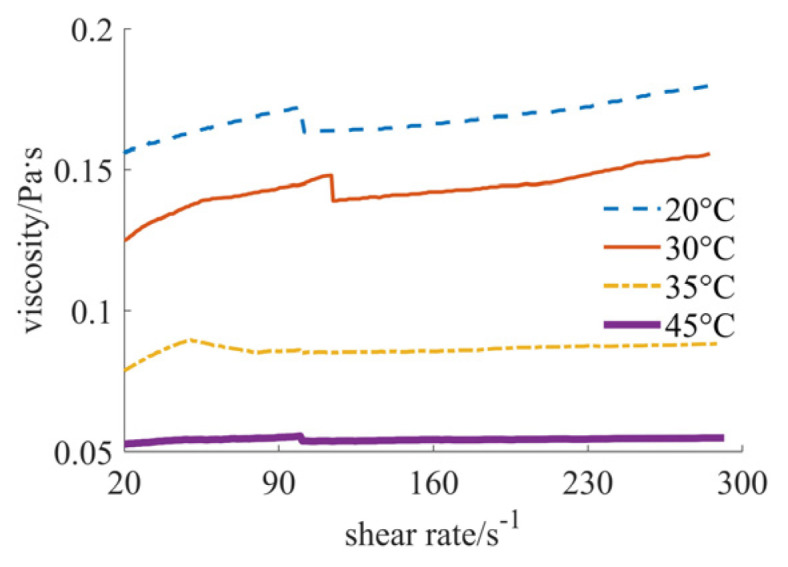
Viscosity curves of 25% nano and 20% micron case.

Figure 4, Figure 5 and Figure 6 show the curves of fluid viscosity variation with the shear rate at different temperatures when the solid phase material is a nano-sized and micron-sized silica mixed powder. In Figure 4, the proportion of the mixed materials is 40% nano-sized and 5% micron-sized silicon dioxide powders. In Figure 5, the mixed proportion is 30% nano-sized and 15% micron-sized silicon dioxide powders. In Figure 6, the results for the 25% nano-sized and 20% micron-sized silicon dioxide powders are provided. The rheological properties of the fluid are significantly different when the mixing proportions of nano-sized and micron-sized powders are changed. When the solid phase material is 40% nano-sized and 5% micron-sized silicon dioxide powder, the temperature is 20 °C and 30 °C, and the shear rate is less than 200 s^−1^, the fluid shows the characteristics of shear thinning, and when the shear rate is larger than 200 s^−1^, the fluid reflects the characteristics of shear thickening; when the temperature is 35 °C and 45 °C, the fluid exhibits the characteristics of completely shear thinning. When the solid phase material is 25% nano-sized and 20% micron-sized silicon dioxide powder, the fluid reflects the characteristics of shear thickening at four different temperatures. When the solid phase material is 30% nano-sized and 15% micron-sized silica powder and the temperature is 20 °C and 30 °C, the fluid shows the characteristics of completely shear thickening. When the temperature is 35 °C and 45 °C, the fluid shows the characteristics of shear thickening when the shear rate is small, and the fluid shows the characteristics of Newtonian fluid when the shear rate is large. By comparing Figure 5 and Figure 6, it can be found that the shear thickening degree of the fluid in Figure 6 is significantly lower than that in Figure 5. By comprehensively comparing the viscosity values, it can be seen that the higher the added proportion of micron-sized silica powder, the smaller the viscosity value.

By comprehensively analyzing Figure 1, Figure 2, Figure 3, Figure 4, Figure 5 and Figure 6, it can be seen that at the same temperature, the rheological properties and viscosity values are significantly different for different powder particle sizes. The larger the particle size of the solid phase powder material, the smaller the viscosity value of the fluid. The comprehensive comparison is shown in Table 1, where the viscosity range and rheological behavior are provided. It can be observed that the viscosity value of the final mixed slurry is generally required to be lower than 3 Pa·s, which can meet the characteristics of layer-by-layer spreading and printing [11]. The viscosity values obtained from the experimental results all meet this requirement. Because the required viscosity value is small, the fluid shows the characteristics of Newtonian fluid or shear thinning, indicating a better alignment with the requirements.

## 4. Rheological Predicted Model

### 4.1. POA-DELM

The pelican optimization algorithm-deep extreme learning machine (POA-DELM) is used to establish a rheological predicted model, introduced in detail below.

#### 4.1.1. Pelican Optimization Algorithm (POA)

The POA is proposed by Trojovsky et al., based on pelican hunting behavior [21]. The detailed process is introduced below. First, the pelican population is initialized randomly, following the methods of Refs. [22,23],

(1) $$h_{i_{1},j_{1}}=l_{j_{1}}+rand(u_{j_{1}}-l_{j_{1}})$$

where *i*_1_ = 1, 2, …, *N*, *j*_1_ = 1, 2, …, *m*, *h_i_*_1, *j*1_ is the *j*1th dimension position of the *i*1th pelican; rand is the random number in the range of [0, 1]; *u_j_*_1_ and *l_j_*_1_ represent the upper and lower bounds. The pelican population can be expressed by the matrix as follows,

(2) $$H={\left[\begin{array}{c} H_{1}\\ \vdots \\ H_{i_{1}}\\ \vdots \\ H_{N} \end{array}\right]}_{N\times m}=\left[\begin{array}{ccccc} h_{1,1} & \cdots & h_{1,j_{1}} & \cdots & h_{1,m}\\ \vdots & \ddots & \vdots & & \vdots \\ h_{i_{1},1} & \cdots & h_{i_{1},j_{1}} & \cdots & h_{i_{1},m}\\ \vdots & & \vdots & \ddots & \vdots \\ h_{N,1} & \cdots & h_{N,j_{1}} & \cdots & h_{N,m} \end{array}\right]$$

where ***H*** is the pelican population matrix, and *H_i_*_1_ is the position of the *i*1th pelican. The target function of the pelican is defined as follows,

(3) $$F={\left[\begin{array}{c} F_{1}\\ \vdots \\ F_{i_{1}}\\ \vdots \\ F_{N} \end{array}\right]}_{N\times 1}={\left[\begin{array}{c} F(H_{1})\\ \vdots \\ F(H_{i_{1}})\\ \vdots \\ F(H_{N}) \end{array}\right]}_{N\times 1}$$

where *F_i_*_1_ is the target value of the *i*1th pelican, and *F_i_*_1_ = *F*(*H_i_*_1_).

Second, the pelican starts to update its position. The updating process can be divided into two stages: prospecting and developing.

(1) Prospecting stage. The pelican determines the position of the prey and then moves towards this determined area, expressed as follows:

(4) $$h_{i_{1},j_{1}}^{q_{1}}=\{\begin{array}{c} h_{i_{1},j_{1}}+rand(p_{j_{1}}-D\times h_{i_{1},j_{1}}),\;F_{p}<F_{i_{1}}\\ h_{i_{1},j_{1}}+rand(h_{i_{1},j_{1}}-p_{j_{1}}),\;otherwise \end{array}$$

where $h_{i_{1},j_{1}}^{q_{1}}$ is the new position of the *i*1th pelican at the *j*1th dimension in this stage; D is a random number of 1 or 2; *p_j_*_1_ is the position of the prey at the *j*1th dimension; *F_p_* is the target value of the prey. If the new position can improve the target value, it is accepted. The detailed expression is as follows:

(5) $$H_{i_{1}}=\{\begin{array}{c} H_{i_{1}}^{q_{1}},\;F_{i_{1}}^{q_{1}}<F_{i_{1}}\\ H_{i_{1}},\;otherwise \end{array}$$

where $H_{i_{1}}^{q_{1}}$ is the new position of the *i*1th pelican; $F_{i_{1}}^{q_{1}}$ is the target value after the update at this stage.

(2) Developing stage. When the pelican is hunting the prey, the expression is defined as follows:

(6) $$h_{i_{1},j_{1}}^{q_{2}}=h_{i_{1},j_{1}}+R(1-\frac{t}{t_{\max}})(2\times rand-1)\times h_{i_{1},j_{1}}$$

where $h_{i_{1},j_{1}}^{q_{2}}$ is the new position of the *i*1th pelican at the *j*1th dimension in this stage; *R* is a constant, which is equal to 0.2; *t* and *t*_max_ are the current and the maximum iteration times. In this stage, the position of the pelican is updated in a manner similar to that used in Equation (5), which is defined in detail as follows:

(7) $$H_{i_{1}}=\{\begin{array}{c} H_{i_{1}}^{q_{2}},\;F_{i_{1}}^{q_{2}}<F_{i_{1}}\\ H_{i_{1}},\;otherwise \end{array}$$

where $H_{i_{1}}^{q_{2}}$ is the new position of the *i*1th pelican; $F_{i_{1}}^{q_{2}}$ is the target value after the update at the second stage.

#### 4.1.2. Deep Extreme Learning Machine (DELM)

ELM (extreme learning machine) [24,25] is defined as a single hidden-layer feedforward neural network, which is unique in that it randomly initializes the connection weights and biases from the input layer to the hidden layer and then directly determines the connection weights from the output layer to the hidden layer through analytical solutions. The design enables ELM to work at high speeds during training, while demonstrating excellent performance in regards to numerous practical problems.

**Figure 7:**
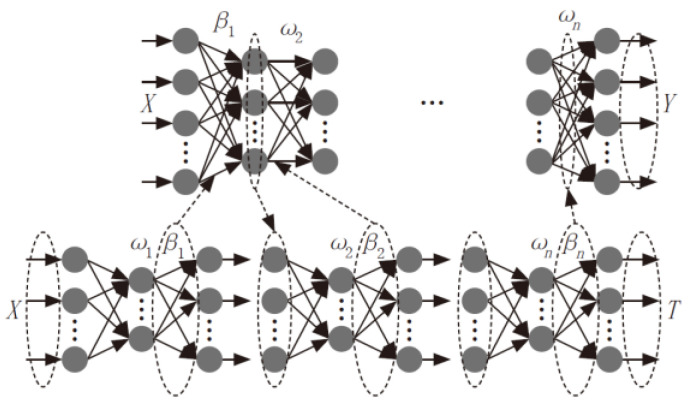
DELM training process.

Autoencoder (AE) is an unsupervised learning method that, after training, can copy inputs to outputs. The training of AE is also unsupervised. Therefore, applying the idea of AE to extreme learning machines can be seen as an approximator, making the input and output of the network model consistent. Due to the powerful feature representation ability of ELM-AE, it is used as the basic unit of DELM. Similar to traditional deep learning algorithms, DELM initializes the input weights of each hidden layer through ELM-AE and implements hierarchical unsupervised learning. However, this method is significantly different from traditional deep learning algorithms, as DELM does not require reverse fine-tuning. By minimizing reconstruction errors, DELM allows the output to be infinitely close to the original input. After training at each layer, DELM can learn advanced features of the original data. Figure 7 shows the model training process of DELM [26].

For equal dimensional feature representation, the output weight of the DELM hidden layer is as follows:

(8) $$\beta =TG^{-1}$$

where *T* is the output of the output layer; *G* is the output of the hidden layer. For dimensionality reduction and high-dimensional expression, the output weight of the DELM hidden layer is as follows:

(9) $$\beta ={\left(\frac{E}{D^{\prime }}+G^{T}G\right)}^{-1}GTX$$

where *D’* is the regularization parameter; *β* is the output weight; *β* = [*β*_1_, *β*_2_, …, *β_n_*]; E is the identity matrix; *X* is the input data; *X* = [*X*_1_, *X*_2_, …, *X_n_*].

#### 4.1.3. The Detailed Process of POA-DELM

Randomly initializing the input layer weights in ELM-AE without updating them during training directly leads to unstable DELM performance and reduces network performance. The prediction accuracy of the network can be effectively improved by optimizing the DELM weight parameters using the POA algorithm. The detailed process is expressed as follows:

(1) Set the parameters for the POA, including population size *N*, which is set as 20; the maximum number of iterations *t*_max_, which is set as 100; and the upper and lower bound [*lb*, *ub*], which is [−1, 1].

(2) Perform population initialization, combining ownership values as the position of each pelican, initializing the position of individual pelicans in the population using Equation (1).

(3) Using the mean square error between the actual and predicted values as the target function, calculate and rank the target values of the individual pelicans and find the one with the lowest fitness value as the optimal pelican individual.

(4) Update the location and target value of the pelican population during the exploration and development phase. Compare the current iteration with the optimal individual from the previous iteration. If it is better than the last generation, update the position of the optimal individual; otherwise, it remains unchanged.

(5) Determine whether the maximum number of iterations has been reached. If so, end and input the optimal weight combination into the DELM; otherwise, proceed back to Step (3).

### 4.2. Training Results

The data from the viscosity measurement is divided into training and test sets, based on 80% and 20% proportions. Then, they are introduced into the above POA-DELM algorithm to build a prediction model, which is then compared with the fitted and traditional rheological models.

**Figure 8:**
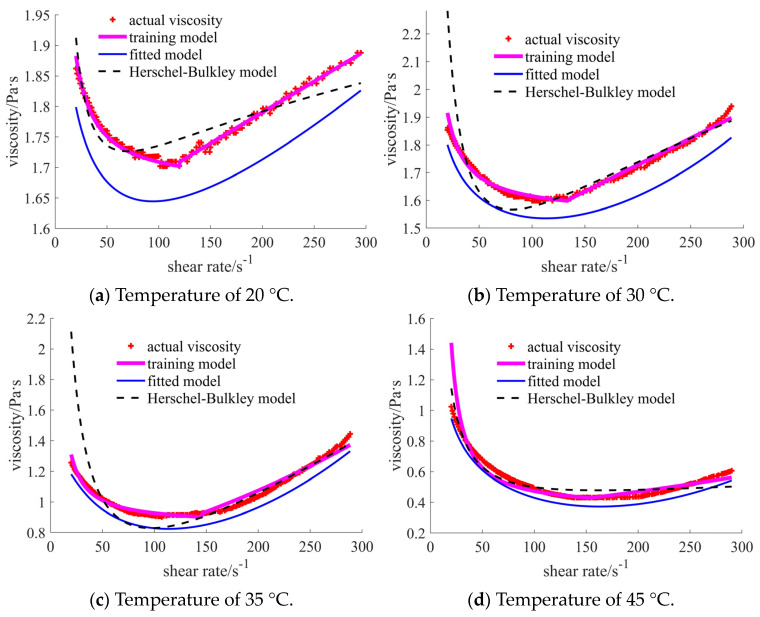
Comparison of the viscosities for the nano-sized case under different temperatures.

For case 1, the comparisons in Figure 8 show that the training model coincides well with the actual points. The curves of the commonly used traditional models (such as the Herschel–Bulkley model) show obvious differences in certain shear rate ranges. It is noted that the detailed traditional and the subsequent fitted models are provided in Appendix A. For the case of 20 °C, the error becomes larger when the shear rate reaches 70 s^−1^. In the range of [70, 200] s^−1^, the Herschel–Bulkley viscosities are larger than those in the actual data, while they become smaller when the shear rate exceeds 200 s^−1^. When the temperature is 30 °C, the main difference concerns the small shear rate area, especially when the shear rate is lower than 45 s^−1^. A similar situation occurs in the case of the 35 °C temperature. In the case of the 45 °C temperature, the bigger error occurs in the initial stage of the shear rate for the training model when compared with the results for the Herschel–Bulkley model. However, the training curve is closer to the actual point when the shear rate exceeds 40 s^−1^. The common polynomial is assumed to be the target function when conducting self-fitting. The curves of the self-fitted models are located below the actual points in all four cases.

In Table 2, the common statistical results are listed. Root mean square error (RMSE) is used to judge the error between the observed and actual values [27]. Mean absolute percentage error (MAPE) is used to judge the mean value of the percentage error between the observed and actual values [28]. Mean absolute error (MAE) can well reflect the exact error of the model [27]. The values are expected to be as small as possible for the above indexes. The last index is R-squared (R^2^), used to judge the goodness of the model fitting [29]. The numbers 1, 2, and 3 represent the results of the prediction, fitted, and traditional rheological models, respectively. When the number is closer to 1, the model is better fitted. The training models in the cases of 20 °C, 30 °C, and 35 °C temperatures reflect the best results for all statistical indexes. In the case of 45 °C, the MAPE and MAE are the smallest, while the R^2^ is not ideal for the training model. The results shown in the table validate the phenomenon that appears in the curve comparison figures.

**Figure 9:**
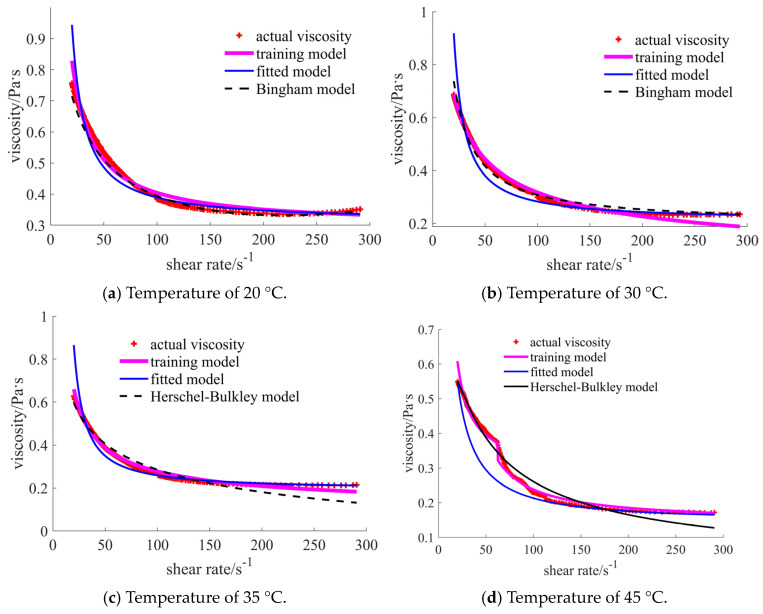
Comparison of the viscosities for sub-micron-sized case under different temperatures.

For case 2, the comparisons are shown in Figure 9, and the pink and black curves are more coincident with the actual points than is the blue curve. The fitted model presents a larger error at the lower shear rate. It is noted that the final case is not smooth, and the training process is divided into three parts. In the lower temperature cases, the Bingham model is more suitable than the Herschel–Bulkley model, while both show the yielding of fluids.

The statistical results shown in Table 3 further demonstrate the phenomenon exhibited in Figure 5. The results show that the training model is the most promising for each case.

**Figure 10:**
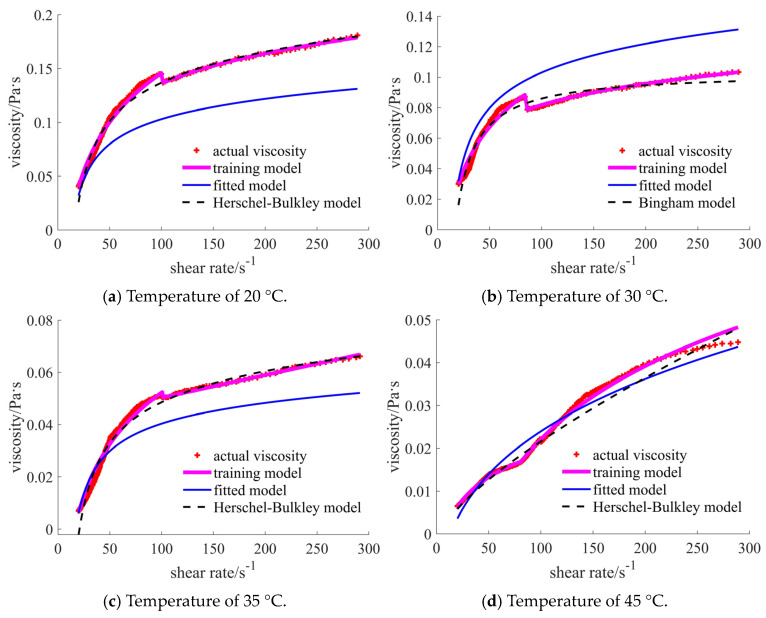
Comparison of the viscosities for micron-sized case under different temperatures.

The comparisons for case 3 are given in Figure 10, which show that the training model coincides more with the actual results than with those of the traditional rheological model. The main reason for this may be the sudden viscosity change at a certain shear rate, which causes a larger error. The fitted model is the poorest in most cases, except for in the final case. A large error occurs in the initial and final shear rate ranges for the fitted and traditional rheology models.

The statistical results are given in Table 4, demonstrating the effectiveness of the training model. The small relative coefficient factor further illustrates the fact that the fitted model is not recommended.

**Figure 11:**
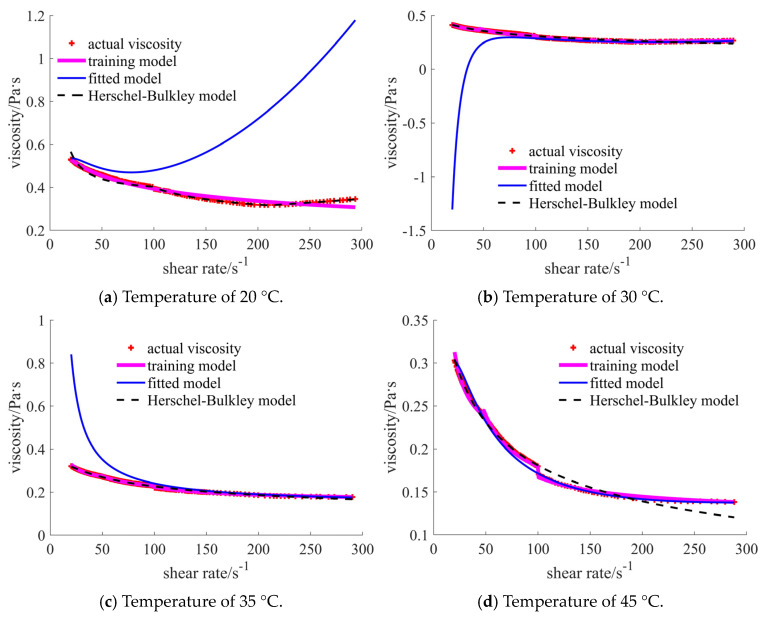
Comparison of the viscosities for 40% nano- and 5% micron-sized case under different temperatures.

The comparisons of case 4 in Figure 11 indicate the obvious error of the fitted model in different shear rate ranges, except for in the final case. Both the training and Herschel–Bulkley models show good coincidence with the actual points.

The error-related factors listed in Table 5 illustrate that the training model obtains a smaller RMSE, MAPE, and MAE and a larger R^2^ compared to the results for the traditional Herschel–Bulkley model.

**Figure 12:**
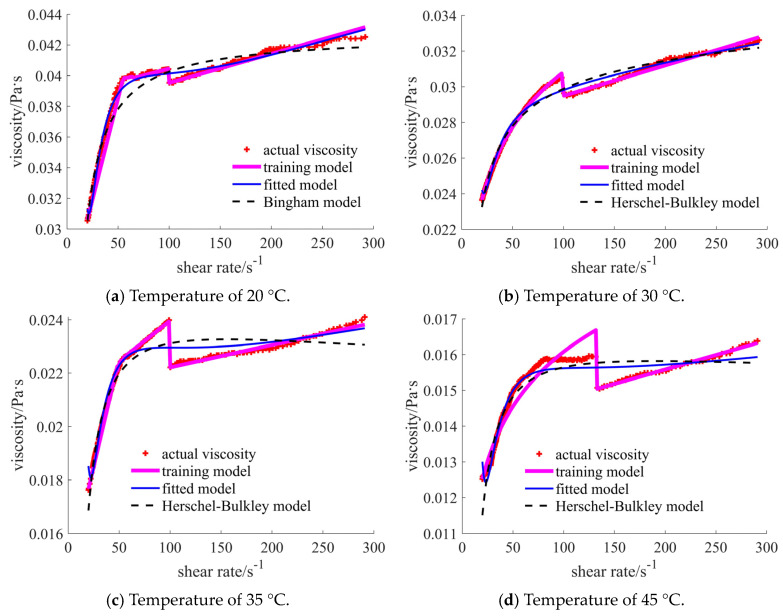
Comparison of the viscosities for 25% nano- and 20% micron-sized case under different temperatures.

In case 5, Figure 12 shows that the sudden decrease in the viscosity occurs at the shear rate of 105 s^−1^, which causes the change in the trends of the fitted and Herschel–Bulkley curves. The small resolution of the viscosity at the *y*-axis enlarges the decrease, when compared with that in Figure 5.

The statistical results in Table 6 show that the R^2^ of each model is larger than 0.9, which indicates that all models can express the actual viscosity well. However, the prediction model presents the largest R^2^ and the smallest error.

**Figure 13:**
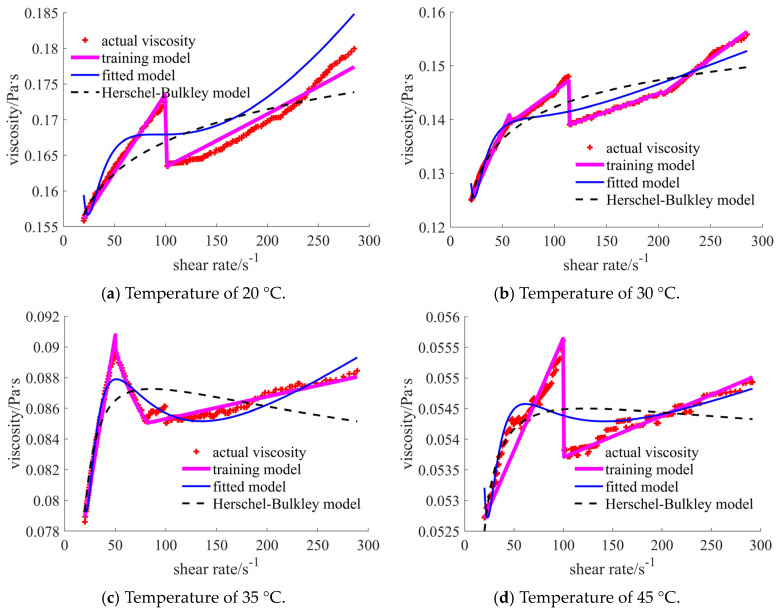
Comparison of the viscosities for 30% nano- and 15% micron-sized case under different temperatures.

The curves for case 6 are shown in Figure 13. The obvious fluctuation of the actual viscosity points occurs in each case. The traditional rheological curves are lower, then higher, and finally lower when compared with the actual points, which is in poorer accordance with the actual points than is the case for the fitted models. The curve of the prediction model is closest to the red points in the tables.

In Table 7, the prediction model results are the most appropriate, followed by the fitted models, which exhibit better results do than the traditional rheological models in the case of 30 °C, 35 °C, and 45 °C temperatures.

## 5. Discussion

The rheological behavior of silica resin-based slurry is investigated using different powder diameters and temperatures. The obvious non-Newtonian behavior is presented in each case, which is similar to the research of Corcione, C. [30], in which the commercial epoxy-based resin SL5170 was mixed with 50% loaded silica (average particle size 5 μm). The viscosity decreases with the increase in the shear rate. The differences are reflected in the monomer, particle size, and solid load, leading to the similar tendency of the curve and different viscosity values. The viscosity will increase along with the increase in the number of functional groups of the monomer. Ozkan, B. et al. found that when preparing the silica slurry, the number of functional groups has an important effect on the refractive index, thus affecting the viscosity [31]. The 60% solid-loaded SiO_2_ slurry exhibits yielding rheological behavior, which is similar to the results found in the current study. The increased number of functional groups indicates a larger viscosity, and the non-Newtonian behavior is more obvious. Qian, J. M. et al. explored the properties of SiO_2_ slurry with the resins of HDDA, HEA, and TMPTA. When the solid content is 45%, the viscosity is similar to that for the micro-sized powders [32]. Chartier, T. et al. investigated the rheological behavior of micron-sized silica powders mixed with PEAAM, where the solid content is 40 vol%, and the viscosities vary from 1.65 Pa·s to 6.20 Pa·s, depending on the content of the dispersant. When the dispersant (phosphate ester, PE) content equals 5 wt%, the viscosity is higher than that observed in the current work [33]. It is suggested that the choice of resins and dispersants can reduce the viscosity, although the solid content is higher.

According to the results, it is determined that when the powder size is uniform, the larger size will decrease the refractive index gap between the resin and the silica powder, and the viscosity will decrease. In addition, the shear thinning behavior is more obvious in the case of the smaller powder size. Increasing the powder size will transform the shear thinning into shear thickening. When the temperature is considered, the viscosity will decrease along with the temperature increase.

The viscosity curves cannot be expressed using traditional rheological functions, leading to the inaccuracy of fluid dynamic analysis. The POA-DELM algorithm is proposed to build a prediction model. The comparisons indicate that the prediction model is more stable and accurate, especially at the lower and higher shear rates. In some cases, the fluctuation is apparently observed, caused by the extremely small resolution of the *y*-axis.

## 6. Conclusions

The effect of the particle size of solid phase material silica powder on the rheological behavior is studied in regards to ceramic 3D printing. The addition ratio of solid phase material is set to 45%, and the viscosity curve of the slurry with the shear rate is obtained, using a viscometer, at four different temperatures. Based on the experimental results, it can be found that the particle size of silica powder and the experimental temperature exert important effects on the rheological behavior.

When the powder is a uniform single material, the larger the particle size, the lower the viscosity value. When the powder particle size is at the nanoscale, the slurry exhibits two characteristics: a shear thinning at a low shear rate and a shear thickening at a high shear rate. When the powder particle size is at the sub-micron level, the slurry exhibits obvious shear thinning characteristics in the early stage of the shear rate. It exhibits Newtonian fluid-like characteristics in the later stage of the shear rate. The slurry exhibits complete shear thickening characteristics when the powder particle size is at the micrometer level.

When the powder is a mixture of nano- and micro-sized silica materials, the viscosity value gradually decreases with the increase in the proportion of micro-sized powder. The slurry exhibits typical shear thinning characteristics when the proportion of nano-sized powder added is high. The slurry exhibits slight shear thickening characteristics when the proportion of nano-sized powder added is relatively low.

By examining the effect of temperature on the rheological properties of solid powder materials with different particle sizes, it can be observed that at lower temperatures, the viscosity value is relatively high. As the temperature gradually increases, the viscosity value gradually decreases.

The prediction model for the viscosity is built by proposing the POA-DELM algorithm, which can better fit the actual viscosity points, in most cases, than can the self-fitted or the traditional rheological models, allowing for its introduction into the fluid dynamic analysis to obtain more accurate simulation results.

## Figures and Tables

**Table 1:** Comparison results of the viscosities using different powder sizes and temperatures.

	Nano-Sized (Case 1)	Sub-Micron-Sized (Case 2)	Micron-Sized (Case 3)	40% Nano-Sized and 5% Micron-Sized (Case 4)	25% Nano-Sized and 20% Micron-Sized (Case 5)	30% Nano-Sized and 15% Micron-Sized (Case 6)
20 °C	[1.717, 1.888], shear thinning–thickening	[0.351, 0.756], shear thinning	[0.030, 0.103], shear thickening	[0.346, 0.530], shear thinning	[0.031, 0.043], shear thickening	[0.156, 0.180], shear thickening
30 °C	[1.613, 1.939], shear thinning–thickening	[0.235, 0.687], shear thinning	[0.041, 0.181], shear thickening	[0.266, 0.411], shear thinning	[0.024, 0.033], shear thickening	[0.125, 0.156], shear thickening
35 °C	[0.903, 1.445], shear thinning–thickening	[0.215, 0.628], shear thinning	[0.007, 0.066], shear thickening	[0.178, 0.321], shear thinning	[0.017, 0.024], shear thickening	[0.079, 0.088], shear thickening
45 °C	[0.431, 1.024], shear thinning–thickening	[0.172, 0.550], shear thinning	[0.006, 0.045], shear thickening	[0.138, 0.303], shear thinning	[0.012, 0.016], shear thickening	[0.053, 0.055], shear thickening

**Table 2:** Statistical results of nano-sized case under different temperatures.

20 °C					30 °C				
	RMSE	MAPE	MAE	R^2^		RMSE	MAPE	MAE	R^2^
1	0.0057	0.0026	0.0047	0.987	1	0.0131	0.0058	0.0100	0.975
2	0.0736	0.0054	0.0733	0.754	2	0.0836	0.0481	0.0827	0.724
3	0.0217	0.0103	0.0184	0.809	3	0.0891	0.0316	0.0554	0.754
35 °C					45 °C				
	RMSE	MAPE	MAE	R^2^		RMSE	MAPE	MAE	R^2^
1	0.0062	0.0184	0.0196	0.971	1	0.0580	0.0510	0.0323	0.794
2	0.0203	0.0746	0.0784	0.786	2	0.0612	0.1141	0.0610	0.769
3	0.0397	0.0693	0.0751	0.721	3	0.0405	0.0650	0.0347	0.859

**Table 3:** Statistical results of sub-micron-sized case under different temperatures.

20 °C					30 °C				
	RMSE	MAPE	MAE	R^2^		RMSE	MAPE	MAE	R^2^
1	0.0203	0.0347	0.0171	0.975	1	0.0129	0.0309	0.0095	0.993
2	0.0412	0.0533	0.0293	0.896	2	0.0576	0.0796	0.0393	0.852
3	0.0227	0.0312	0.0178	0.968	3	0.0170	0.0369	0.0148	0.987
35 °C					45 °C				
	RMSE	MAPE	MAE	R^2^		RMSE	MAPE	MAE	R^2^
1	0.0104	0.0262	0.0082	0.992	1	0.0134	0.0304	0.0100	0.988
2	0.0439	0.0665	0.0282	0.865	2	0.0729	0.1502	0.0600	0.636
3	0.0240	0.0632	0.0189	0.960	3	0.0176	0.0537	0.0139	0.979

**Table 4:** Statistical results of micron-sized case under different temperatures.

20 °C					30 °C				
	RMSE	MAPE	MAE	R^2^		RMSE	MAPE	MAE	R^2^
1	0.0034	0.0377	0.0028	0.994	1	0.0032	0.0514	0.0024	0.979
2	0.0262	0.1803	0.0212	0.604	2	0.0168	0.2536	0.0155	0.433
3	0.0045	0.0462	0.0036	0.988	3	0.0039	0.0602	0.0032	0.969
35 °C					45 °C				
	RMSE	MAPE	MAE	R^2^		RMSE	MAPE	MAE	R^2^
1	0.0021	0.0800	0.0016	0.986	1	0.0006	0.0193	0.0004	0.997
2	0.0076	0.1857	0.0066	0.813	2	0.0021	0.0976	0.0018	0.963
3	0.0025	0.1009	0.0020	0.980	3	0.0018	0.0648	0.0014	0.972

**Table 5:** Statistical results of 40% nano- and 5% micron-sized case under different temperatures.

20 °C					30 °C				
	RMSE	MAPE	MAE	R^2^		RMSE	MAPE	MAE	R^2^
1	0.0087	0.0146	0.0053	0.984	1	0.0034	0.0064	0.0022	0.996
2	0.2371	0.3770	0.1319	0.714	2	0.4359	0.5432	0.2118	0.358
3	0.0126	0.0205	0.0097	0.965	3	0.0081	0.0222	0.0064	0.976
35°C					45°C				
	RMSE	MAPE	MAE	R^2^		RMSE	MAPE	MAE	R^2^
1	0.0016	0.0050	0.0013	0.998	1	0.0024	0.0090	0.0020	0.998
2	0.1432	0.3228	0.0928	0.675	2	0.0059	0.0216	0.0050	0.984
3	0.0026	0.0081	0.0018	0.996	3	0.0044	0.0160	0.0029	0.992

**Table 6:** Statistical results of 25% nano- and 20% micron-sized case under different temperatures.

20 °C					30 °C				
	RMSE	MAPE	MAE	R^2^		RMSE	MAPE	MAE	R^2^
1	0.0003	0.0067	0.0002	0.989	1	0.0001	0.0031	0.0001	0.998
2	0.0005	0.0087	0.0003	0.968	2	0.0002	0.0075	0.0002	0.988
3	0.0006	0.0133	0.0005	0.954	3	0.0003	0.0092	0.0003	0.983
35 °C					45 °C				
	RMSE	MAPE	MAE	R^2^		RMSE	MAPE	MAE	R^2^
1	0.0001	0.0056	0.0001	0.991	1	0.0002	0.0104	0.0001	0.963
2	0.0003	0.0126	0.0003	0.957	2	0.0003	0.0138	0.0002	0.940
3	0.0004	0.0169	0.0004	0.929	3	0.0003	0.0157	0.0002	0.926

**Table 7:** Statistical results of 30% nano- and 15% micron-sized case under different temperatures.

20 °C					30 °C				
	RMSE	MAPE	MAE	R^2^		RMSE	MAPE	MAE	R^2^
1	0.0009	0.0045	0.0008	0.976	1	0.0005	0.0029	0.0004	0.995
2	0.0030	0.0158	0.0027	0.744	2	0.0021	0.0121	0.0017	0.925
3	0.0027	0.0129	0.0022	0.784	3	0.0026	0.0140	0.0020	0.887
35 °C					45 °C				
	RMSE	MAPE	MAE	R^2^		RMSE	MAPE	MAE	R^2^
1	0.0003	0.0034	0.0003	0.978	1	0.0002	0.0027	0.0001	0.883
2	0.0006	0.0057	0.0005	0.935	2	0.0003	0.0043	0.0002	0.715
3	0.0015	0.0152	0.0013	0.613	3	0.0004	0.0055	0.0003	0.602

## Data Availability

The original contributions presented in the study are included in the article, further inquiries can be directed to the corresponding authors.

## Appendix A

Case 1 (20 °C):

Fitted model: $\mu =2.506{\dot{\gamma }}^{-0.1168}-0.05298{\dot{\gamma }}^{-1}+0.001819\dot{\gamma }$

Herschel–Bulkley model: $\mu =\frac{10.0305}{\dot{\gamma }}+1.0766{\dot{\gamma }}^{0.0908}$

Case 1 (30 °C):

Fitted model: $\mu =2.237{\dot{\gamma }}^{-0.0932}+2.163{\dot{\gamma }}^{-1}+0.000006{\dot{\gamma }}^{2}$

Herschel–Bulkley model: $\mu =\frac{30.5844}{\dot{\gamma }}+0.2940{\dot{\gamma }}^{0.3180}$

Case 1 (35 °C):

Fitted model: $\mu =7.331{\dot{\gamma }}^{-0.4672}-12.65{\dot{\gamma }}^{-1}+0.00001029{\dot{\gamma }}^{2}$

Herschel–Bulkley model: $\mu =\frac{40.3677}{\dot{\gamma }}+0.0041{\dot{\gamma }}^{1.0086}$

Case 1 (45 °C):

Fitted model: $\mu =-0.1775{\dot{\gamma }}^{0.3357}+5.987{\dot{\gamma }}^{-1}+0.000006886{\dot{\gamma }}^{2}+1.134$

Herschel–Bulkley model: $\mu =\frac{19.4158}{\dot{\gamma }}+0.0657{\dot{\gamma }}^{0.3339}$

Case 2 (20 °C):

Fitted model: $\mu =104.1{\dot{\gamma }}^{-0.953}-109.7{\dot{\gamma }}^{-1}-0.0003286\dot{\gamma }+0.000001613{\dot{\gamma }}^{2}+0.2152$

Bingham model: $\mu =\frac{10.6792}{\dot{\gamma }}+0.2975$

Case 2 (30 °C):

Fitted model: $\mu =2.9512{\dot{\gamma }}^{-0.4844}-14322{\dot{\gamma }}^{-6.4805}$

Bingham model: $\mu =\frac{10.8790}{\dot{\gamma }}+0.199$

Case 2 (35 °C):

Fitted model: $\mu =23.7267{\dot{\gamma }}^{0.0007}+2.2444x^{-0.1947}-24.4338$

Herschel–Bulkley model: $\mu =\frac{7.0324}{\dot{\gamma }}+0.6515{\dot{\gamma }}^{-0.2491}$

Case 2 (45 °C):

Fitted model: $\mu =0.091{\dot{\gamma }}^{0.07}+8.772{\dot{\gamma }}^{-1}$

Herschel–Bulkley model: $\mu =\frac{-118.3552}{\dot{\gamma }}+105.3416{\dot{\gamma }}^{-0.9314}$

Case 3 (20 °C):

Fitted model: $\mu =0.0283{\dot{\gamma }}^{0.1836}-0.5082{\dot{\gamma }}^{-1}$

Bingham model: $\mu =\frac{-1.8606}{\dot{\gamma }}+0.0712{\dot{\gamma }}^{0.1697}$

Case 3 (30 °C):

Fitted model: $\mu =0.05557{\dot{\gamma }}^{0.1569}-1.144{\dot{\gamma }}^{-1}$

Herschel–Bulkley model: $\mu =\frac{-1.7561}{\dot{\gamma }}+0.1035$

Case 3 (35 °C):

Fitted model: $\mu =0.02391{\dot{\gamma }}^{0.1443}-0.6098{\dot{\gamma }}^{-1}$

Herschel–Bulkley model: $\mu =\frac{-0.9134}{\dot{\gamma }}+0.0261{\dot{\gamma }}^{0.1723}$

Case 3 (45 °C):

Fitted model: $\mu =0.4327{\dot{\gamma }}^{0.1708}-0.4474{\dot{\gamma }}^{0.158}$

Herschel–Bulkley model: $\mu =\frac{-0.0153}{\dot{\gamma }}+0.0007{\dot{\gamma }}^{0.7464}$

Case 4 (20 °C):

Fitted model: $\mu =4.796{\dot{\gamma }}^{-0.5004}-10.81{\dot{\gamma }}^{-1}+0.00001086{\dot{\gamma }}^{2}$

Herschel–Bulkley model: $\mu =\frac{-1.7242}{\dot{\gamma }}+1.3086{\dot{\gamma }}^{-0.2516}$

Case 4 (30 °C):

Fitted model: $\mu =0.0004383{\dot{\gamma }}^{1.032}+36.05{\dot{\gamma }}^{-1}-1251{\dot{\gamma }}^{-2}+0.0001382{\dot{\gamma }}^{-3}$

Herschel–Bulkley model: $\mu =\frac{-1.6581}{\dot{\gamma }}+1.0916{\dot{\gamma }}^{-0.2640}$

Case 4 (35 °C):

Fitted model: $\mu =0.118{\dot{\gamma }}^{0.02947}+10.38{\dot{\gamma }}^{-1}-2.14{\dot{\gamma }}^{-2}+1663{\dot{\gamma }}^{-3}$

Herschel–Bulkley model: $\mu =\frac{-1.9623}{\dot{\gamma }}+1.1139{\dot{\gamma }}^{-0.3278}$

Case 4 (45 °C):

Fitted model: $\mu =0.008946{\dot{\gamma }}^{0.417}+13.03{\dot{\gamma }}^{-1}-209.6{\dot{\gamma }}^{-2}+1154{\dot{\gamma }}^{-3}$

Herschel–Bulkley model: $\mu =\frac{-1.7776}{\dot{\gamma }}+1.4060{\dot{\gamma }}^{-0.4250}$

Case 5 (20 °C):

Fitted model: $\mu =0.0090{\dot{\gamma }}^{0.2543}+1.611{\dot{\gamma }}^{-1}-55.6345{\dot{\gamma }}^{-2}+564.687{\dot{\gamma }}^{-3}$

Bingham model: $\mu =\frac{-0.2442}{\dot{\gamma }}+0.0427$

Case 5 (30 °C):

Fitted model: $\mu =0.0137{\dot{\gamma }}^{0.1434}+0.5037{\dot{\gamma }}^{-1}-19.5352{\dot{\gamma }}^{-2}+214.5925{\dot{\gamma }}^{-3}$

Herschel–Bulkley model: $\mu =\frac{-0.1102}{\dot{\gamma }}+0.0250{\dot{\gamma }}^{0.0466}$

Case 5 (35 °C):

Fitted model: $\mu =0.0070{\dot{\gamma }}^{0.1941}+0.87{\dot{\gamma }}^{-1}-31.9645{\dot{\gamma }}^{-2}+338.9710{\dot{\gamma }}^{-3}$

Herschel–Bulkley model: $\mu =\frac{-0.2150}{\dot{\gamma }}+0.0327{\dot{\gamma }}^{-0.056}$

Case 5 (45 °C):

Fitted model: $\mu =0.0065{\dot{\gamma }}^{0.1409}+0.4960{\dot{\gamma }}^{-1}-20.0505{\dot{\gamma }}^{-2}+227.0217{\dot{\gamma }}^{-3}$

Herschel–Bulkley model: $\mu =\frac{-0.1324}{\dot{\gamma }}+0.0206{\dot{\gamma }}^{-0.0421}$

Case 6 (20 °C):

Fitted model: $\mu =4.786{\dot{\gamma }}^{-0.606}+76.7539{\dot{\gamma }}^{-2}-16.3508{\dot{\gamma }}^{-1}+0.0003\dot{\gamma }$

Herschel–Bulkley model: $\mu =\frac{-0.0043}{\dot{\gamma }}+0.1397{\dot{\gamma }}^{0.0387}$

Case 6 (30 °C):

Fitted model: $\mu =0.0334{\dot{\gamma }}^{0.2502}+4.8859{\dot{\gamma }}^{-1}-150.9113{\dot{\gamma }}^{-2}+1523{\dot{\gamma }}^{-3}$

Herschel–Bulkley model: $\mu =\frac{-0.2145}{\dot{\gamma }}+0.1207{\dot{\gamma }}^{0.039}$

Case 6 (35 °C):

Fitted model: $\mu =0.0153{\dot{\gamma }}^{0.2855}+3.8961{\dot{\gamma }}^{-1}-112.9258{\dot{\gamma }}^{-2}+1049.8{\dot{\gamma }}^{-3}$

Herschel–Bulkley model: $\mu =\frac{-0.3838}{\dot{\gamma }}+0.1139{\dot{\gamma }}^{-0.0486}$

Case 6 (45 °C):

Fitted model: $\mu =0.0349{\dot{\gamma }}^{0.0713}+0.8169{\dot{\gamma }}^{-1}-25.0061{\dot{\gamma }}^{-2}+253.4879{\dot{\gamma }}^{-3}$

Herschel–Bulkley model: $\mu =\frac{-0.0744}{\dot{\gamma }}+0.0581{\dot{\gamma }}^{-0.011}$

## References

[1] Jiang Y.Z., Wang Y.L., Lichade K., He H., Feinerman A., Pan Y. (2020). Textured window design for continuous projection stereolithography process. Manuf. Lett..

[2] Im Y.G., Chuang S.I., Son J.H., Jung Y., Jo J., Jeong H. (2002). Functional prototype development: Inner visible multi-color prototype fabrication process using stereo lithography. J. Mater. Process. Technol..

[3] Kim Y.J., Kim H.N., Kim D. (2024). A study on effects of curing and machining conditions in post-processing of SLA additive manufactured polymer. J. Manuf. Process..

[4] Halloran J.W. (2016). Ceramic Stereolithography: Additive Manufacturing for Ceramics by Photopolymerization. Annu. Rev. Mater. Res..

[5] Smirnov A., Tikhonov A., Dubinin O., Chugunov S., Shishkovsky I. (2022). Piezoelectric properties of the 3D-printed lead-free ceramics. Ferroelectrics.

[6] Li X., Su H.J., Dong D., Zhao D., Liu Y., Shen Z., Jiang H., Guo Y., Liu H., Fan G. (2022). Enhanced comprehensive properties of stereolithography 3D printed alumina ceramic cores with high porosities by a powder gradation design. J. Mater. Sci. Technol..

[7] Ding G.J., He R.J., Zhang K.Q., Xia M., Feng C., Fang D. (2020). Dispersion and stability of SiC ceramic slurry for stereolithography. Ceram. Int..

[8] Wu X.Q., Xu C.J., Zhang Z.M. (2021). Preparation and optimization of Si3N4 ceramic slurry for low-cost LCD mask stereolithography. Ceram. Int..

[9] Tang J., Chang H.T., Guo X.T., Liu M., Wei Y., Huang Z., Yang Y. (2022). Preparation of photosensitive SiO2/SiC ceramic slurry with high solid content for stereolithography. Ceram. Int..

[10] Zhang K.Q., Meng Q.Y., Zhang X.Q., Qu Z.L., Jing S.K., He R.J. (2021). Roles of solid loading in stereolithography additive manufacturing of ZrO2 ceramic. Int. J. Refract. Met. Hard Mater..

[11] Li K.H., Zhao Z. (2017). The effect of the surfactants on the formulation of UV-curable SLA alumina suspension. Ceram. Int..

[12] Xing H.Y., Zou B., Lai Q.G., Huang C., Chen Q., Fu X., Shi Z. (2018). Preparation and characterization of UV curable Al2O3 suspensions applying for stereolithography 3D printing ceramic microcomponent. Powder Technol..

[13] Li X.B., Zhang J.X., Duan Y.S., Liu N., Jiang J., Ma R., Xi H., Li X. (2020). Rheology and Curability Characterization of Photosensitive Slurries for 3D Printing of Si3N4 Ceramics. Appl. Sci..

[14] Wu W.W., Deng X., Ding S., Zhang Y., Liu D., Zhang J. (2023). Investigation of Obstacles with Interactive Elements on the Flow in SiC Three-Dimensional Printing. 3D Print. Addit. Manuf..

[15] Wu W.W., Deng X., Ding S., Zhu L., Wei X., Song A. (2021). Effect mechanism of multiple obstacles on non-Newtonian flow in ceramic 3D printing (arcuate elements). Ceram. Int..

[16] Wu W.W., Deng X., Ding S., Zhang Y., Tang B., Shi B. (2023). Micro-flow investigation on laying process in Al2O3 stereolithography forming. Phys. Fluids.

[17] Zhang K.X., Liu B.S., Li T., Luo G., Li S., Duan W., Wang G. (2023). Simulation and experimental analysis on the deformation rate on slender rod parts during the recoating process in high viscosity ceramic stereolithography. Int. J. Adv. Manuf. Technol..

[18] He L., Song X. (2018). Supportability of a High-Yield-Stress Slurry in a New Stereolithography-Based Ceramic Fabrication Process. JOM.

[19] Cheng S.J., Qin X.B., Bian M.F., Wang Z. (2003). Study on liquid structure and viscosity of In-5%Cu alloy. Rare Met. Mater. Eng..

[20] Lin C., Wu S.S., Lv S.L., Zeng J., An P. (2019). Viscosity variation of hypereutectic Al-Si alloys with high iron contents around liquidus temperature. Int. J. Cast Met. Res..

[21] Trojovsky P., Dehghani M. (2022). Pelican Optimization Algorithm: A Novel Nature-Inspired Algorithm for Engineering Applications. Sensors.

[22] Kusuma P.D., Dinimaharawati A. (2022). Hybrid Pelican Komodo Algorithm. Int. J. Adv. Comput. Sci. Appl..

[23] Li Y.T., Liu Y., Lin C.L., Wen J., Yan P., Wang Y. (2023). An Improved Pelican Optimization Algorithm Based on Chaos Mapping Factor. Eng. Lett..

[24] Huang G.B., Zhu Q.Y., Siew C.K. (2006). Extreme learning machine: Theory and applications. Neurocomputing.

[25] Huang G.B., Zhou H.M., Ding X.J., Zhang R. (2012). Extreme Learning Machine for Regression and Multiclass Classification. IEEE Trans. Syst. Man Cybern. Part B-Cybern..

[26] Tang J.X., Deng C.W., Huang G.B., Zhao B. (2015). Compressed-Domain Ship Detection on Spaceborne Optical Image Using Deep Neural Network and Extreme Learning Machine. IEEE Trans. Geosci. Remote Sens..

[27] Willmott C.J., Matsuura K. (2005). Advantages of the mean absolute error (MAE) over the root mean square error (RMSE) in assessing average model performance. Clim. Res..

[28] De Myttenaere A., Golden B., Le Grand B., Rossi F. (2016). Mean absolute percentage error for regression models. Neurocomputing.

[29] Chicco D., Warrens M.J., Jurman G. (2021). The coefficient of determination R-squared is more informative than SMAPE, MAE, MAPE, MSE and RMSE in regression analysis evaluation. Peerj Comput. Sci..

[30] Corcione C., Greco A., Montagna F., Licciulli A., Maffezzoli A. (2005). Silica moulds built by stereolithography. J. Mater. Sci..

[31] Ozkan B., Sameni F., Bianchi F., Zarezadeh H., Karmel S., Engstrøm D.S., Sabet E. (2022). 3D printing ceramic cores for investment casting of turbine blades, using LCD screen printers: The mixture design and characterisation. J. Eur. Ceram. Soc..

[32] Qian J., Sun B., Zhao Q., Sun J., Zhang H. (2022). Study on Rheology and Stability of Light Curing Silicon Oxide Ceramic Slurry. Advances in Machinery, Materials Science and Engineering Application.

[33] Chartier T., Badev A., Abouliatim Y., Lebaudy P., Lecamp L. (2012). Stereolithography process: Influence of the rheology of silica suspensions and of the medium on polymerization kinetics–cured depth and width. J. Eur. Ceram. Soc..

